# Perspective of a Pediatrician: Shared Pathogenesis of the Three Most Successful Pathogens of Children

**DOI:** 10.3389/fcimb.2020.585791

**Published:** 2020-10-15

**Authors:** Elaine I. Tuomanen

**Affiliations:** Department of Infectious Diseases, St Jude Children’s Research Hospital, Memphis, TN, United States

**Keywords:** pneumococcus, meningococcus, haemophilus, PAF receptor, laminin receptor

## Abstract

Highly successful invasive pathogens exploit host vulnerabilities by adapting tools to co-opt highly conserved host features. This is especially true when pathogens develop ligands to hijack trafficking routes or signaling patterns of host receptors. In this context, highly successful pathogens can be grouped together by the patterns of organs infected and diseases they cause. In the case of this perspective, the focus is on the historically most successful invasive bacterial pathogens of children that cause pneumonia, sepsis and meningitis: *Streptococcus pneumoniae, Haemophilus influenzae*, and *Neisseria meningitidis*. This triad shares a ligand to bind to PAF receptor to enter host cells despite early defenses by innate immunity. All three also target laminin receptor to cross endothelial barriers using a common set of molecular tools that may prove to be a design for a cross-protective vaccine.

## Introduction

“*The challenge is to figure out why the most virulent bacterial pathogens of children cause the same pattern of disease, particularly meningitis, and share the same unusual microbial physiology, particularly autolysis and natural transformation*”.

Joshua Lederberg, PhD

Nobel Laureate

Personal communication

Every pediatrician will tell you that, historically, the major invasive pathogens of children that they dread the most are *Streptococcus pneumoniae, Haemophilus influenzae*, and *Neisseria meningitidis* (Kim, 2010; Lundbo and Benfield, 2017). They share striking features of the pattern of disease. All three most commonly attack children under the age of 5 years. All three begin infection by asymptomatic carriage in the nasopharynx, spread through the respiratory tract, multiply quickly to high titer bacteremia and cross the blood brain barrier to cause meningitis (Loughren et al., 2019). It is the final step to meningitis that truly sets these three apart and begs the question, what do they “know” about host vulnerability that promotes a course of infection that is so glaringly lethal? What unusual features of microbial physiology relate to shared pathogenesis? Several major surface features that promote virulence differ between them and thus, are not likely to explain the shared organ tropism. Their capsules serve to protect all three of these bacteria from phagocytosis but are of widely varying chemical composition. *Haemophilus* and meningococcus are Gram negative and thus have a thin cell wall and an outer membrane, while the Gram positive pneumococcus has a thick cell wall and no outer membrane. Determining why this seemingly mixed triad causes such a similar pattern of disease is a significant challenge that is only partially solved.

## Carriage

Pneumococcus, *Haemophilus* and meningococcus circulate in the population by asymptomatic carriage in the nasopharynx of young children (Adegbola et al., 2014). Their mechanisms of attachment are diverse, and each has several ligand receptor interactions with the nasopharyngeal mucosa. During multiple events of carriage in early childhood, the host acquires immunity to the dominant capsular antigens and a variety of surface proteins which, in most cases, appears to be enough to limit further invasion (Segal and Pollard, 2005; Ramos-Sevillano et al., 2019). Unencapsulated strains colonize the mucosa of the upper respiratory tract quite well. The multiplicity of adherence events for each pathogen complicates the design of simple protein-based vaccines to eliminate carriage as a first step in defense. Clearly, these three pathogens start at the same physical point of entry to the host using a very different set of capabilities. Then the story changes.

## Shared Invasion Strategy 1: Enter Cells Despite Innate Immunity

**Figure 1:**
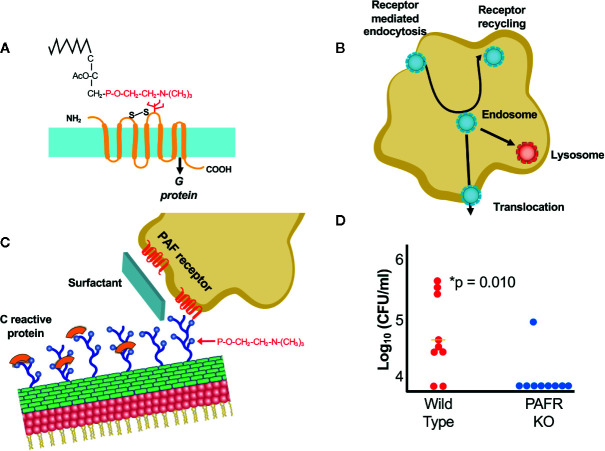
Interactions of bacteria with ChoP-PAFR. **(A)** Platelet activating factor, an inflammatory lipid chemokine. The portion in red is ChoP which is present on bacterial surfaces while the black is the lipid backbone of PAF that is missing in the bacterial form. The 7 transmembrane PAF receptor (PAFR) is shown in yellow. **(B)** Trafficking of PAFR upon ligation by ChoP is diagrammed. Receptor mediated endocytosis engulfs the PAFR bound vesicle containing bacteria into the host cell cytoplasm. The endosome then may traffic to the lysosome for killing, recycle back to the cell surface or translocate across the cell barrier in the process of invasion. **(C)** ChoP (blue balls on cell wall) binds PAFR (red transmembrane lines on host cell). The innate immune system counteracts ChoP by C-reactive protein (orange caps covering ChoP), surfactant (broad sheet of secretions rich in ChoP) and anti-ChoP antibodies. **(D)** Mice were challenged with pneumococci intravenously and the presence bacteria in the cerebrospinal fluid was quantified at 6 h. Animals lacking PAFR (blue dots) are protected from meningitis compared to wild type (WT, red dots) (adapted from Radin et al., 2005).

Infection of the lower respiratory tract is the go/no go for invasive disease. While these bacteria use several different ligand/receptor interactions at any one site, it is in the respiratory tract that the three pathogens reveal they also harbor a shared invasion strategy that is effective despite innate immunity. All three bacteria decorate their surfaces with the small molecule phosphorylcholine (ChoP) (**Figure 1A**) that is added to bacterial surface components by the shared LicD protein, a ChoP transferase (Weiser et al., 1997; Weiser et al., 1998a; Zhang et al., 1999). First described for the pneumococcus, ChoP is covalently added to the teichoic acid and lipoteichoic acid of the cell wall (Briles and Tomasz, 1973). As a key bioactive adduct, ChoP on the pneumococcal cell wall serves as a non-covalent docking station for over a dozen secreted choline binding proteins that are interchanged to modulate contact interactions between the pathogen and host (Gosink et al., 2000). Rather than being added to cell wall, ChoP appears on the lipopolysaccharide of *Haemophilus* (Weiser et al., 1997) and on meningococcal lipopolysaccharide and pili (Weiser et al., 1998a). Further work has expanded the list of pathogens that display ChoP to incorporate most pulmonary pathogens, including *Pseudomonas, Klebsiella, Legionella* and even mycoplasma (Clark and Weiser, 2013). Further underlining the breadth of use of this determinant, virtually all oral commensals display it on their surfaces (Gillespie et al., 1993).

### The ChoP Disguise Promotes Bacterial Entry Into Cells

Expression of ChoP on the bacterial surface mimics the critical chemical determinant of the lipid chemokine platelet activating factor (PAF) (**Figure 1A**) (Cundell et al., 1995; Swords et al., 2000; Iuchi et al., 2019). The binding of PAF-ChoP to PAF receptor (PAFR) results in two outcomes: 1) it induces multiple inflammatory signals transduced by coupled G-proteins in platelets, macrophages, epithelial and endothelial cells (Chao and Olson, 1993; Izumi and Shimizu, 1995; Honda et al., 2002); and 2) the triad of invasive pathogens uses ChoP to co-opt PAFR trafficking whereby the receptor undergoes rapid internalization independent of G-protein activation (**Figure 1B**) (Chen et al., 2002; Fillon et al., 2006). Uptake of the chemokine or the bacteria by PAFR is followed by either trafficking to the lysosome *via* Rab5 and Rab7 or recycling to the cell surface (Ishii et al., 1998; Chen et al., 2002). This recirculation of PAFR from the host cell surface to the cytoplasm and back to the surface provides a shuttle for adherent bacteria to enter epithelial and endothelial cells *via* receptor mediated endocytosis (Ring et al., 1998). This trafficking involves co-localization of bacteria, PAFR and the scaffold protein *ß*-arrestin (Luttrell and Lefkowitz, 2002; Spiegel, 2003; Radin et al., 2005; Iovino et al., 2013). Bacteria in the intracellular vacuole are then subject to three fates: being killed in the lysosome, transcytosing across the cell to exit the basal surface, or recycling back to the apical surface (Ring et al., 1998).

### The Host Fights Back With the Innate Immune Response

The innate immune response strongly counteracts ChoP mediated interactions between bacteria and host cells (Gould and Weiser, 2002) (**Figure 1C**). The ChoP decoration is the target of natural antibodies that are present at birth even without prior bacterial challenge (Lieberman et al., 1974; Goldenberg et al., 2004). ChoP is the determinant recognized by the first responder of the innate immune system, C-reactive protein, which by binding ChoP, serves as a competitive inhibitor (Weiser et al., 1998b; Clark and Weiser, 2013; Langereis et al., 2019). Furthermore, the lung is awash in ChoP as a major component of surfactant (Gould and Weiser, 2002). Thus, early events in establishing pneumonia are played out by tipping the balance between host recognition of ChoP as a foreign disguise on bacteria and fighting back *vs* falling for the deception that ChoP-coated bacteria mimic the beneficial proinflammatory cytokine PAF.

### When Is ChoP-PAFR Operative in Infection?

Presentation of ChoP on the surfaces of all three pathogens is phase variable with greater abundance correlating with greater interactions with mucosal cells and decreased expression characterizing sustained circulation in the bloodstream (Weiser et al., 1994; Weiser et al., 1997; Serino and Virji, 2002). ChoP-PAFR is not the only mechanism of cellular entry for the three major pathogens, but it is a shared one of importance as shown by the failure of mice lacking PAFR to rapidly spread infection between organs (Rijneveld et al., 2004; Radin et al., 2005). These animals show a delayed translocation of bacteria from lung to blood and a significant defect in causing meningitis (**Figure 1D**).

Recently, evidence indicates that bacterial surface components, free from the intact bacterium, also transit barriers using the ChoP tag. Bacterial surface components are released upon lysis of bacteria by antibiotics. Using the pneumococcal cell wall as an example, ChoP on the teichoic acid enables cell wall fragments in blood to bind to PAFR on vascular endothelial cells and traffic into the brain and heart (Tuomanen et al., 1985; Fillon et al., 2006). These cell wall components are recognized by Toll-like receptor 2 and thus, are highly inflammatory (Yoshimura et al., 1999). Upon entering the brain parenchyma, ChoP cell wall induces caspase-dependent apoptosis of neurons (Braun et al., 1999; Orihuela et al., 2006). In the heart, ChoP-bearing cell wall induces death of cardiomyocytes and lethal cardiac dysfunction (Fillon et al., 2006).

In the specific clinical context of a pregnant mouse being treated for bacteremic pneumonia with antibiotics, cell wall released in the bloodstream crosses the placenta and enters the fetal brain. The interaction required for translocation of cell wall across the placenta is ChoP binding to PAFR (Humann et al., 2016). It is not as yet known if components from other bacteria decorated with ChoP also cross the placenta. While cell wall is highly inflammatory in most models, the interaction of cell wall components with embryonic neurons appears to be fundamentally different than the catastrophic death of postnatal neurons. Early in development of the fetal neocortex, neuronal progenitor cells bearing TLR2 respond to cell wall by enhancing proliferation without any cell death (Humann et al., 2016). This results in a larger pool of progenitors that constitute a wave of excess cells that migrates through all the cortical layers resulting in a 50% increase in total cell number in the neocortex. The cortical layers form normally but each layer has an abnormally high number of cells, an aberration in brain architecture that persists after birth. A bigger brain is not always a better brain. Mice born after experiencing a cell wall-induced proliferative wave of neurons during gestation exhibit abnormal social behavior, cognitive deficits and permanent changes in cortical architecture (Humann et al., 2016).

## Shared Invasion Strategy 2: Co-opt Receptor Mediated Endocytosis to Cross Cell Barriers

Having passed from the lung into blood, the three pathogens undergo phase variation to increase capsule thickness and downregulate ChoP to effectively avoid phagocytosis resulting in high titer bacteremia, a prerequisite explaining their particular propensity to invade other organs and cause sepsis (Orihuela et al., 2003). Bacterial titers above 10e5 cfu/ml of blood trigger translocation across substantial vascular barriers in the heart and the brain. While the three major meningeal pathogens use several strategies to transit both through and between endothelial cells, they all share the ability to exploit laminin receptor mediated endocytosis that enables entry into the brain and heart.

**Figure 2:**
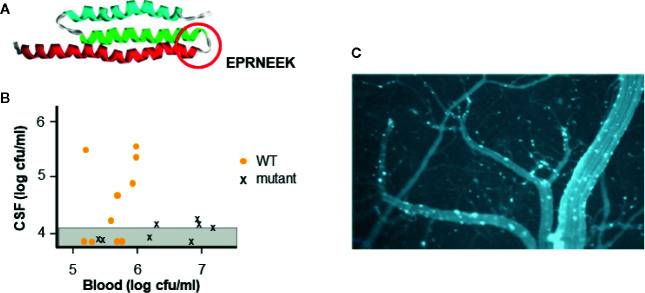
Interaction of CbpA-like adhesin with laminin receptor. **(A)** The structure of the CbpA adhesin domain highlighting the amino acid sequence of the region required for binding to the blood brain barrier (circle) (adapted from Luo et al., 2005). **(B)** Pneumococcal mutant lacking CbpA is compared to wild type (WT) for causing meningitis. Strains were injected intravenously into mice (each symbol is one mouse) and the presence of bacteria in the blood and CSF was determined. Graph shows that WT pneumococci cross the blood brain barrier into the CSF at a blood threshold of ~10e5 cfu. Pneumococcal mutants lacking CbpA do not cross the blood brain barrier into the CSF even at 10e7 cfu in the blood. (adapted from Orihuela et al., 2003) **(C)** Beads coated with CbpA and injected intravenously in mice, adhere to cerebral capillaries (white) and cross into the brain parenchyma (dark) as viewed through a cranial window of a mouse. (adapted from Orihuela et al., 2009).

All three pathogens harbor a functional homolog of the pneumococcal adhesin CbpA that binds to laminin receptor of the host (**Figure 2A**) (Orihuela et al., 2003). Within the CbpA domain that binds laminin receptor, the sequence EPRNEEK forms a loop between two helices (Luo et al., 2005). Both the sequence and tertiary structure are highly conserved among pneumococci and are critical to function (Mann et al., 2014); pneumococci lacking CbpA show poor penetration into the cerebrospinal fluid in mouse models (**Figure 2B**). Although there is no sequence homology to this domain in their genomes, meningococci display pilus protein PilQ and outer membrane protein PorA and *Haemophilus* present membrane protein OmpP2 that crossreact with antibodies to CbpA and enable both bacteria to also bind to laminin receptor (Mann et al., 2014). This binding facilitates both adherence of bacteria to the cerebral vasculature and subsequent translocation across the endothelial cell cytoplasm into the brain parenchyma. Intravenous injection of CbpA-coated beads into mice followed by imaging of the brain surface through a cranial window dramatically reveals the ability of CbpA to bring particles to and through the cerebral capillary endothelium (**Figure 2C**).

Further impact of the adhesin/laminin receptor interaction is revealed by recent work that, during the course of bacteremic pneumonia, the pneumococcus can translocate into myocardial cells forming microlesions (Brown et al., 2014). It appears that the mechanism of translocation across the cardiac vascular endothelium again involves CbpA/laminin receptor facilitating live bacterial entry into cardiomyocytes. Cardiac damage is reflected by increased levels of Troponin T and abnormal electrocardiography as the bacterial microcolonies interrupt electrical transduction pathways. Necroptosis and apoptosis within the lesions lead to permanent scar formation and contribute to cardiac morbidity and mortality, underappreciated as sequela of clinical pneumonia (Reyes et al., 2017). Such cardiac injury has been associated clinically with the acute and convalescent phases of pneumonia but the mechanism of this link is only now appreciated.

### Design of a Cross-Protective Vaccine

Cross-reactivity between the meningeal pathogens of the ligands for laminin receptor-mediated translocation is highlighted by the observation that induction of antibody by vaccination with CbpA conveys protection not only against pneumococcal infection but also against *Haemophilus* sepsis and otitis media and meningococcal meningitis in mouse models (Mann et al., 2014; Rowe et al., 2019). A CbpA-based vaccine is effective in preventing cardiac lesions based on blocking the shared CbpA/laminin receptor mechanism (Mann et al., 2014). Just as was the case for ChoP/PAF receptor as a generalized code for pulmonary/meningeal pathogens entering cells, CbpA/laminin receptor is a shared key to recognizing the blood brain barrier and the heart. Neurotropic pathogens as disparate as syphilis, Venezuelan equine encephalitis virus, Sinbis virus, and prions all share entry into the central nervous system *via* laminin receptor. If many laminin receptor ligands also cross react, this is a feature that could be exploited for a more broadly protective meningitis vaccine; how broadly protective is yet to be determined.

## Summary

Pathogens utilize a vast array of individual host/receptor interactions. However, particularly successful ones, like pneumococcus, *Haemophilus influenzae*, and meningococcus, they harbor a uniquely effective shared invasion strategy targeting receptor mediated endocytosis by PAFR and laminin receptor. These mechanisms to cross endothelial barriers have proven successful in causing a similar pattern of severe infections including sepsis and meningitis. Using this commonality to design a vaccine to elicit crossreactive antibodies against the bacterial ligands for laminin receptor may prove to be a broadly effective counterattack.

## Author Contributions

ET conceived of the concept and wrote the manuscript.

## Funding

This research was supported by NIH grant NIAID R01 AI 128756 and ALSAC. The content is solely the responsibility of the authors and does not necessarily represent the official views of the National Institutes of Health.

## Conflict of Interest

The author declares that the research was conducted in the absence of any commercial or financial relationships that could be construed as a potential conflict of interest.
